# Whole body hyperthermia, but not skin hyperthermia, accelerates brain and locomotor limb circulatory strain and impairs exercise capacity in humans

**DOI:** 10.14814/phy2.13108

**Published:** 2017-01-20

**Authors:** Steven J. Trangmar, Scott T. Chiesa, Kameljit K. Kalsi, Niels H. Secher, José González‐Alonso

**Affiliations:** ^1^ Centre for Human Performance, Exercise and RehabilitationBrunel University LondonUxbridgeUnited Kingdom; ^2^ The Copenhagen Muscle Research CentreDepartment of Anaesthesia, RigshospitaletUniversity of CopenhagenCopenhagenDenmark; ^3^ Present address: Department of Life SciencesUniversity of RoehamptonWhitelands CollegeHolybourne AvenueLondonSW15 4JDUnited Kingdom; ^4^ Present address: Institute of Cardiovascular ScienceUniversity College London1 St. Martin's Le Grand, London, EC1A 4NPUnited Kingdom

**Keywords:** Hyperthermia, maximal exercise, regional blood flow and metabolism

## Abstract

Cardiovascular strain and hyperthermia are thought to be important factors limiting exercise capacity in heat‐stressed humans, however, the contribution of elevations in skin (*T*_sk_) versus whole body temperatures on exercise capacity has not been characterized. To ascertain their relationships with exercise capacity, blood temperature (*T*_B_), oxygen uptake (*V̇*O_2_), brain perfusion (MCA *V*_mean_), locomotor limb hemodynamics, and hematological parameters were assessed during incremental cycling exercise with elevated skin (mild hyperthermia; HYP_mild_), combined core and skin temperatures (moderate hyperthermia; HYP_mod_), and under control conditions. Both hyperthermic conditions increased *T*_sk_ versus control (6.2 ± 0.2°C; *P *<* *0.001), however, only HYP_mod_ increased resting *T*_B_, leg blood flow and cardiac output (*Q̇*), but not MCA *V*_mean_. Throughout exercise, *T*_sk_ remained elevated in both hyperthermic conditions, whereas only *T*_B_ was greater in HYP_mod_. At exhaustion, oxygen uptake and exercise capacity were reduced in HYP_mod_ in association with lower leg blood flow, MCA *V*_mean_ and mean arterial pressure (MAP), but similar maximal heart rate and *T*_B_. The attenuated brain and leg perfusion with hyperthermia was associated with a plateau in MCA and two‐legged vascular conductance (VC). Mechanistically, the falling MCA VC was coupled to reductions in *P*aCO_2_, whereas the plateau in leg vascular conductance was related to markedly elevated plasma [NA] and a plateau in plasma ATP. These findings reveal that whole‐body hyperthermia, but not skin hyperthermia, compromises exercise capacity in heat‐stressed humans through the early attenuation of brain and active muscle blood flow.

## Introduction

Aerobic exercise performance (Dill et al. 1931; Galloway and Maughan 1997; Tatterson et al. 2000; Ely et al. 2010; Périard et al. 2011) and maximal aerobic power (*V̇*O_2max_) (Pirnay et al. 1970; Sawka et al. 1985; Nybo et al. 2001; González‐Alonso and Calbet 2003; Arngrímsson et al. 2004; Lafrenz et al. 2008) are degraded by heat stress. The precise impairment in *V̇*O_2max_ has been controversial, with some observing little (≤3%) or no reduction (Williams et al. 1962; Rowell et al. 1966; Klausen et al. 1967), and others reporting more substantial decrements (~ 7–30%) (Sawka et al. 1985; Nybo et al. 2001); the largest decline observed when internal and skin temperatures are markedly elevated prior to exercise (Pirnay et al. 1970; Arngrímsson et al. 2004). Conversely, brief exposure to heat stress, inducing skin hyperthermia in the absence of internal/core hyperthermia, does not appear to substantially reduce *V̇*O_2max_ (Arngrímsson et al. 2004). The combined thermal stress of high core and skin temperature, therefore, appears to be a prerequisite for a compromised maximal aerobic capacity. There is, however, surprisingly limited information on the precise mechanisms underpinning the hyperthermia‐induced suppression of *V̇*O_2max_ and, in particular, the cardiovascular adjustments to incremental exercise with different extents of skin and whole‐body hyperthermia.

It has been hypothesized that elevations in skin temperature, by requiring a large proportion of the cardiovascular capacity, are the primary factor leading to a compromised maximal aerobic performance. This concept was substantiated by the classical observation that, despite a small, nonsignificant fall in *V̇*O_2max_ (~ 3%, *N* = 6), cardiac output (*Q̇*) is reduced during the high‐intensity stages of graded exercise in the heat, compared with a temperate environment, in untrained men (Rowell et al. 1966). This reduction in *Q̇* could conceivably impair O_2_ delivery to the active skeletal muscle; however, there is some evidence that *Q̇* is higher during the early stages of intense constant‐load exercise with body hyperthermia in trained individuals (González‐Alonso and Calbet 2003; González‐Alonso et al. 2004). Therefore, it is unlikely that high skin blood flow requirements per se compromise systemic perfusion. On the other hand, restrictions in active skeletal muscle perfusion may play an important role in the reduced aerobic capacity in hyperthermic conditions. Under control (normothermic) conditions, skeletal muscle O_2_ delivery is tightly coupled to the metabolic demand during submaximal exercise (Andersen and Saltin 1985; Delp and Laughlin 1998; Saltin et al. 1998; González‐Alonso et al. 2002; Delp and O'Leary 2004); regulation that is lost at high intensities as, prior to volitional exhaustion, systemic and active skeletal muscle (in addition to brain and respiratory muscle) blood flow becomes restricted (González‐Alonso and Calbet 2003; Mortensen et al. 2005, 2008; Vogiatzis et al. 2009; Calbet et al. 2015). The attenuated limb blood flow per unit of power when approaching maximal exercise intensities occurs concomitantly with enhanced local vasoconstrictor activity and reductions in stroke volume (González‐Alonso and Calbet 2003; Calbet et al. 2007; Mortensen et al. 2008; Stöhr et al. 2011b; Munch et al. 2014). As a consequence, and in contrast to other important regions of the body such as the brain (Nybo et al. 2002; González‐Alonso et al. 2004; Trangmar et al. 2014), blunted O_2_ delivery may compromise local aerobic metabolism, as maximal skeletal muscle O_2_ extraction is achieved during exhaustive exercise (González‐Alonso and Calbet 2003). Whether the hyperthermia‐related reduction in *V̇*O_2max_ seen during graded exercise to volitional exhaustion is associated with alterations in regional and systemic hemodynamics has never been systematically tested.

The primary aim of this study was to investigate the effect of heat stress, inducing two different grades of hyperthermia, on brain and active limb blood flow and metabolism during incremental cycling exercise to volitional exhaustion. Regional hemodynamics and metabolism during incremental exercise were assessed; (1) after heat exposure sufficient to elevate internal and skin temperature, (2) after a brief heat exposure sufficient to elevate skin temperature and (3) in control conditions. We hypothesized that combined core and skin hyperthermia, but not skin hyperthermia, would compromise *V̇*O_2max_ and exercise capacity in close association with restrictions in brain and active limb perfusion.

## Methods

### Ethical approval

All procedures in this study were approved by the Brunel University London Research Ethics Committee (RE54‐12) and conformed to the guidelines of the World Medical Association (Declaration of Helsinki). All participants provided their oral and written and informed consent prior to participation.

### Participants

Nine healthy male cyclists (mean ± SD; age 26 ± 6 years, stature 181 ± 6 cm, mass 76 ± 9 kg and *V̇*O_2max_ 4.5 ± 0.1 L·min^−1^) participated in the study. Participants arrived at the laboratory postprandial with a normal hydration status and were required to abstain from strenuous exercise and alcohol intake for 24 h and caffeine consumption for 12 h.

### Experimental design

Participants visited the laboratory on three occasions, comprising of a preliminary trial, a hyperthermia trial, and a control trial, each separated by 1 week. The preliminary trial familiarized participants with the testing methodology, prior to performing an incremental exercise test on a cycle ergometer (Lode Excalibur, Groningen, Netherlands) to establish maximal work rate (*W*_max_), maximal heart rate (HR_max_), and *V̇*O_2max_. The initial work rate was equivalent to 50% of predicted *V̇*O_2max_, for 2.5 min, followed by increments of 10% predicted every 2.5 min until the limit of tolerance. Participants were instructed to maintain a cadence between 70 and 90 r.p.m. and the test was terminated when cycling speed dropped below 60 r.p.m. for more than 3 sec, despite strong verbal encouragement to continue. After a 1 h recovery period, participants were dressed in a water‐perfused suit (covering the arms, legs and torso), and laid in a supine position while hot water (50°C) was circulated through, by a temperature controlled water circulator (Julabo F‐34, Seelbach, Germany). A foil blanket, gloves, and hat were worn to minimize heat loss to the environment. After target increases in skin and core temperature (+6 and +1°C, respectively), participants repeated the incremental test to establish HYP_mod_ *W*_max_.

On the hyperthermia trial, participants completed three incremental cycling ergometer exercise tests in the upright position with; (1) HYP_mod_ (with moderate *T*_c_ and high *T*_sk_, after 52 ± 3 min of heat exposure), (2) HYP_mild_ (with a high *T*_sk_ but normal *T*_c_, after 13 ± 1 min of heat exposure) and, (3) control conditions (*T*_a_ 18°C; 36% relative humidity; with fan cooling). On the control trial, the participants completed three incremental cycling ergometer exercise tests in a thermo‐neutral environment (20°C; ≤50% relative humidity; with fan cooling). Each of the incremental cycling tests consisted of 5× ~2.5 min stages at 20, 40, 60, 80, and 100% *W*_max_, and cycling pedal cadence was stable between 70 and 90 r.p.m. On both the hyperthermia and control trials, each incremental test was separated by 1 h of passive recovery while hydration was maintained through the regular consumption of water.

**Figure 1:**
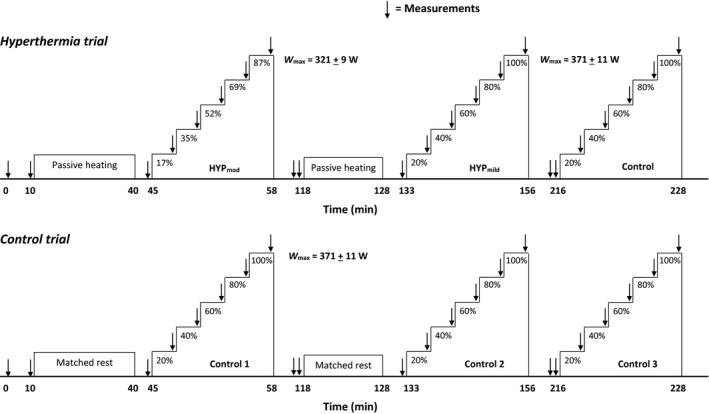
Sequence of the exercise protocols. Participants visited the laboratory on two occasions, with each trial consisting of three incremental cycling exercise tests at intensities relative to *V̇*O_2max_. As HYP_mod_ reduced *V̇*O_2max_ (obtained on the preliminary trial), the absolute work rates of each stage were lower than all other incremental tests (321 ± 9 W vs. 371 ± 11 W). This adjustment allowed for comparisons between incremental tests, relative to *V̇*O_2max_, in either HYP_mod_ or HYP_mild_/control, where the latter two conditions did not reduce *V̇*O_2max_. Passive heating/matched rest durations prior to exercise in HYP_mod_ and HYP_mild_ were 52 ± 3 and 13 ± 1 min, respectively. A minimum of 1 h passive rest separated each incremental exercise bout.

On the hyperthermia trial, brain, active limb and systemic hemodynamics and blood samples from the brachial artery and femoral vein were obtained simultaneously at rest and in the final minute of each exercise stage. Skin and femoral venous temperatures and arterial and femoral venous pressures were recorded continuously. The same measures were collected in the control trial, except for the arterio‐venous blood sampling, leg blood flow (LBF), and blood pressure measurements, and with the addition of esophageal temperature (T_Oes_). Full depiction of the experimental protocol of the study is presented in Figure 1.

### Brain, active limb, and resting systemic hemodynamics

Middle cerebral artery velocity (MCA *V*_mean_) was measured using a 2 MHz pulsed trans‐cranial Doppler ultrasound system (DWL, Sipplingen, Germany). The right MCA was insonated through the temporal ultrasound window, distal to the MCA‐anterior cerebral artery bifurcation, at a depth of 45–60 mm (Aaslid et al. 1982). Regional cerebral (frontal lobe) oxygen saturation (rSO_2_%) was also assessed using near‐infrared spectroscopy (NIRS; INVOS, Somanetics, Troy, MI).

During exercise, LBF was determined using the constant‐infusion thermodilution method (Andersen and Saltin 1985; González‐Alonso et al. 2000). Resting blood flow (*n* = 4) was obtained using duplex Doppler ultrasonography (Vivid 7, Dimension, GE Healthcare, UK), or calculated from the directly obtained a‐vO_2diff_ and estimated leg *V̇*O_2_ (*n* = 5) assuming comparable leg *V̇*O_2_ values than those measured in four participants in this study and previous reports from this laboratory using similar heating protocols (Pearson et al. 2011; Chiesa et al. 2015). *Q̇* at rest was estimated using the Modelflow method (Wesseling et al. 1993), from the directly obtained intra‐arterial pressure wave forms, corrected for age, height, and weight.

### Catheter placement and blood sampling

Participants rested with a slight head‐down tilt while catheters for blood sampling, mean arterial pressure (MAP), femoral venous pressure, and blood temperature were inserted after local anesthesia (1% lidocaine) into the brachial artery of the nondominant arm and anterograde into the right common femoral vein (Logicath Quad lumen, 18 gage, 2.3 mm; MXA234X16X85, Smiths Medical International LTD), the latter using the Seldinger technique. Catheters were inserted by an experienced clinician under ultrasound guidance and were regularly flushed with normal saline (0.9% NaCl) to maintain patency. The time from catheterization to the commencement of resting measurements was ~1 h to allow time for the restoration of normal hemodynamics.

### Blood variables

Arterial and femoral venous blood samples were drawn into preheparinized syringes and analyzed immediately for blood gas variables (ABL 800 FLEX, Radiometer, Copenhagen, Denmark) corrected to blood temperature in the femoral vein. The analyzer was calibrated (one and two‐point) at regular intervals in accordance with manufacturer guidelines. Additional arterial blood samples were collected in 2 mL syringes and transferred to EDTA tubes, centrifuged and separated. Plasma noradrenaline was subsequently determined using an enzyme immunoassay kit (DEE6200, Demeditec Diagnostics GmbH, Kiel, Germany).

### Heart rate, blood pressure and body temperatures

Heart rate was obtained by telemetry (Polar Electro, Kempele, Finland). Arterial and femoral venous pressure waveforms were recorded using pressure transducers (Pressure Monitoring Kit, TruWave, Edwards Lifesciences, Germany) zeroed at the level of the right atrium in the mid‐axillary line (arterial) and at the level of the tip of the catheter (femoral venous). Pressure waveforms were amplified (BP amp, ADIstruments) and sampled at 1000 Hz using a data acquisition unit (Powerlab 16/30, ADInstruments, Oxfordshire, UK) for offline analysis. For measurements of femoral venous blood temperature (*T*_B_), a thermistor (T204a, PhysiTemp, Clifton, NJ) was inserted through the femoral venous catheter and connected to a thermocouple meter (TC‐2000, Sable Systems, NV) and routed through the data acquisition system. In the control trial, esophageal temperature (*T*_Oes_) was measured using a thermistor (Physitemp, New England), inserted pernasally into the esophagus at a depth of ¼ standing height. Increases in core temperature during cycling exercise reflect the rise in femoral venous blood temperature, as *T*_B_ and *T*_Oes_ have been shown to be within ~0.1°C (González‐Alonso et al. 1999). Mean skin temperature (*T*_sk_) from four sites (standard weightings of chest, arm, thigh, and calf; (Ramanathan 1964) was obtained using a wireless monitoring system (iButton^®^, Maxim Integrated, San José, CA).

### Calculations

In the hyperthermia trials, brain and active limb vascular conductance (VC) indices were calculated by dividing MCA *V*_mean_ and LBF (for two‐legged) by perfusion pressure (MAP). Direct measurements of *Q̇* were not possible during exercise, however, to provide some insight into these responses, *Q̇* was calculated using the Fick principle, by estimation of systemic O_2_ extraction from the directly measured limb O_2_ extractions (assuming a linear relationship between these variables, reported in similar exercise protocols; Mortensen et al. 2008; Munch et al. 2014; and accounting for the known reduction in systemic O_2_ extraction with core hyperthermia; González‐Alonso et al. 2004). The following equations were used: *Y* = 1.43X−44.7; *R* ^*2*^ = 0.99; *P > *0.05 for control and HYP_mild_ and *Y* = 1.7322X‐76.126; *R* ^*2*^ = 0.98; *P > *0.05 for HYP_mod_. When leg blood flow measurements were not possible, LBF was calculated from the estimated leg *V̇*O_2_ (assuming that the increase in pulmonary *V̇*O_2_ from baseline reflected only the increase in leg *V̇*O_2_) (Mortensen et al. 2005, 2008; Calbet et al. 2007) and directly measured leg arterial‐to‐femoral venous O_2_ difference.

### Statistics

Differences between exercise conditions were assessed using a two‐way repeated‐measures ANOVA in which condition (Moderate heat stress, mild heat stress, and control) and exercise phase (Rest, 20, 40, 60, 80, and 100%) were the main factors. Where a significant main effect was found, pairwise comparisons were made using the Holm‐Bonferroni procedure. Statistical significance was set at *P *<* *0.05 and all analyses were made using IBM SPSS Statistics (Version 20, IBM Corporation, Armonk, NY, USA).

## Results

### Impact of heat stress and repeated incremental exercise on exercise capacity

On the preliminary visit, heat stress exposure sufficient to induce HYP_mod_ resulted in a reduction in *W*_max_ by ~13 ± 1% (range: 11–17%) and a fall in *V̇*O_2max_ by 8 ± 3% (range 5–12%), despite a similar HR_max_ compared to control. To ensure a comparable percentage of W_max_ across experimental conditions in the subsequent hyperthermia and control trials, the absolute work rates for the incremental stages in HYP_mod_ were reduced by 13 ± 1% (64 ± 2, 128 ± 4, 193 ± 5, 257 ± 7 and 321 ± 9 W) compared to all other incremental tests (74 ± 2, 148 ± 4, 223 ± 7, 297 ± 9 and 371 ± 11 W; Fig. 1).

**Figure 2:**
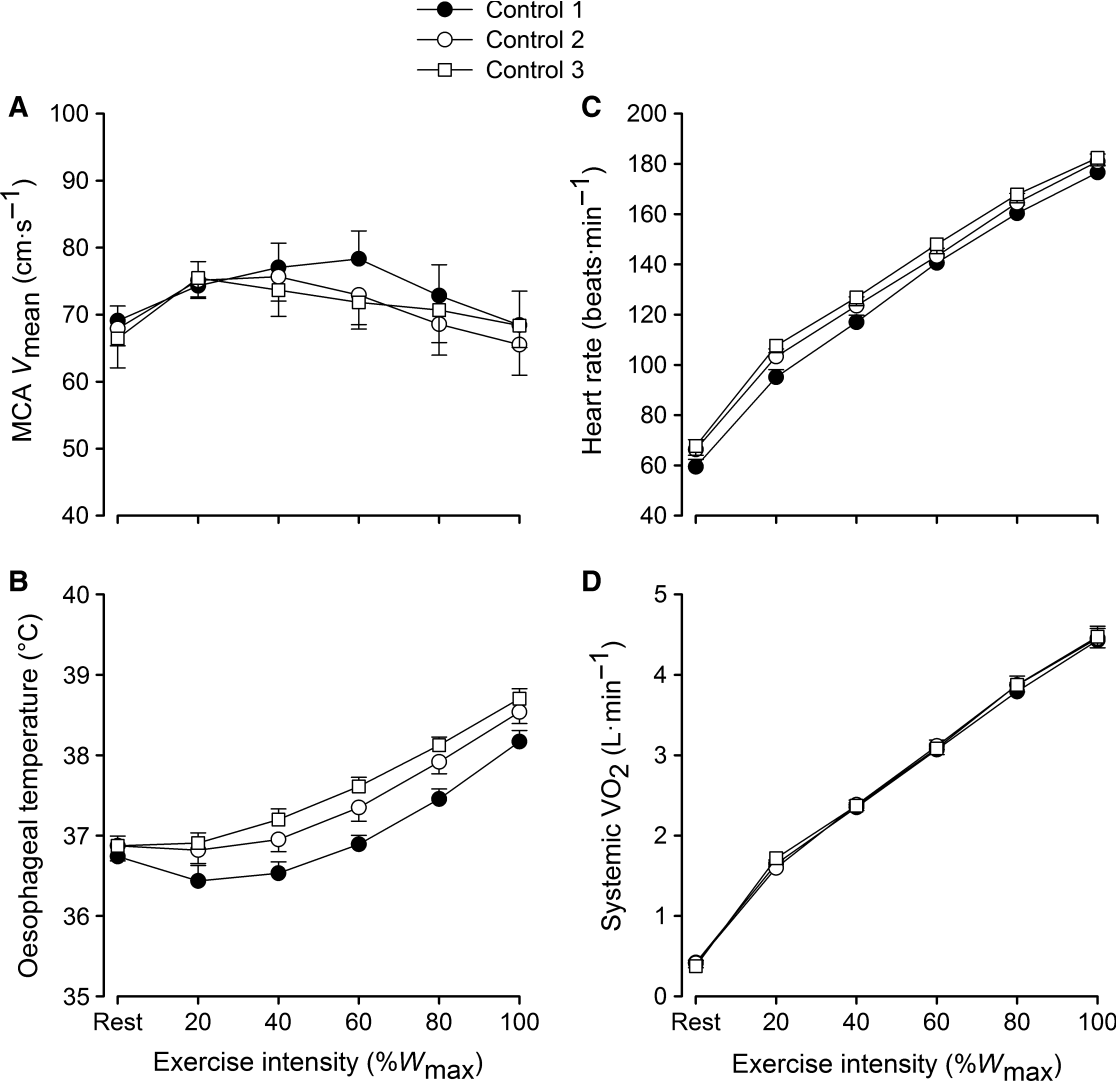
Brain and systemic hemodynamics, and systemic oxygen uptake in response to three incremental exercise bouts on the control trial. Values are means ± SEM for seven participants. Variables in Figure 2B, C, and D increased with exercise intensity (*P *<* *0.01).

During the control trial, where exercise capacity across the three incremental tests was the same, *V̇*O_2max_ (4.4 ± 0.1, 4.5 ± 0.2 and 4.5 ± 0.1 L·min^−1^), HR_max_ (177 ± 3, 181 ± 3, and 182 ± 3 beats·min^−1^), *T*_Oes_ (38.2 ± 0.1, 38.6 ± 0.1, and 38.7 ± 0.1°C) and end‐exercise MCA *V*_mean_ (68 ± 5, 66 ± 5, and 68 ± 3 cm·s^−1^) were not significantly different (Fig. 2). Moreover, the increase in *V̇*O_2_ per unit of power was linear from low to maximal exercise intensities in all three tests (9.2 ± 0.3, 9.5 ± 0.3 and 9.1 ± 0.3 mL.min^−1^.W^−1^; *R* ^2^ = 0.99; *P *<* *0.001). Given the similar exercise capacity, body temperatures and cardio‐respiratory responses to exercise in the control trial, the following sections focus on the effects of temperature manipulation on whole‐body hemodynamics in the hyperthermia trial only.

### Temperature and cardiorespiratory responses to heat stress

**Figure 3:**
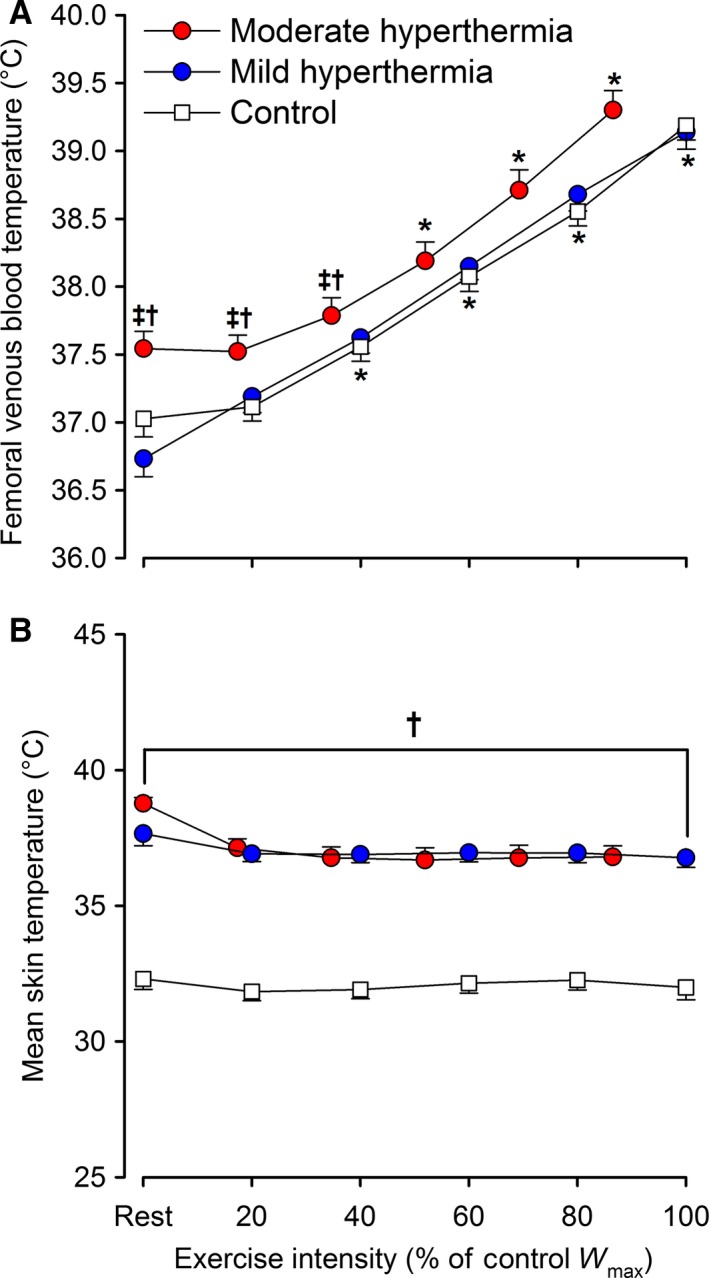
Temperature responses to incremental exercise with different grades of hyperthermia. Femoral venous blood (A) and mean skin (B) temperatures are reported. Values are means±SEM for nine participants. Moderate (internal and skin), mild (skin only) hyperthermia and control exercise are represented. *Different versus rest *P *<* *0.05, ^‡^different versus mild hyperthermia, ^†^different versus control. Presented symbols denote differences between conditions, when compared at a relative % of maximal work rate in hyperthermia or normothermia (100% = 321 W in HYP_mod_ vs. 371 W in HYP_mild_ and control, respectively).

Resting *T*_B_ was elevated in HYP_mod_ compared to HYP_mild_ and control exercise conditions (37.5 ± 0.1 vs. 36.7 ± 0.1 and 37.0 ± 0.1°C; *P* = 0.03), whereas *T*_sk_ was elevated in both heat stress conditions compared to control (~38.2 ± 0.3 vs. 32.3 ± 0.4°C; *P *<* *0.001: Fig. 3A and B). During incremental exercise in HYP_mod_, *T*_B_ was initially unchanged before increasing to a peak of 39.3 ± 0.1°C (*P *<* *0.01 vs. rest), whereas in HYP_mild_ and control, *T*_B_ increased from rest to *W*_max_ (39.1 ± 0.1°C; *P *<* *0.001) and was lower overall compare to HYP_mod_. *T*_sk_ was maintained elevated in both heat stress conditions (~36.9 ± 0.4 vs. 32.0 ± 0.4°C; *P *<* *0.001) and was maintained stable throughout exercise.

Cardiorespiratory variables are presented in Table 1. Briefly, systolic and diastolic blood pressures were lower in HYP_mod_ compared to HYP_mild_ and control (*P *<* *0.001). Respiratory frequency, CO_2_ production (*V̇*CO_2_), and minute ventilation (*V̇*_*E*_) increased with exercise intensity and were lower in HYP_mod_ compared to HYP_mild_ and control (both *P *<* *0.001). End‐tidal *P*O_2_ initially declined before increasing at *W*_max,_ with the reverse response observed for *P*CO_2_; however, there were no differences between the exercise test conditions (*P* = 0.492).

**Table 1:** Cardiorespiratory responses to incremental exercise with different grades of hyperthermia

% of *W*_max_	SBP (mmHg)	DBP (mmHg)	r*f* (breaths·min^−1^)	*V̇*CO_2_ (L·min^−1^)	*V̇*E (L·min^−1^)	PetO_2_ (mmHg)	PetCO_2_ (mmHg)
HYP_mod_							
Rest	138 ± 5^b^	73 ± 2	18 ± 1	464 ± 46	17 ± 2	111 ± 3	33 ± 2
20	157 ± 7^a^	72 ± 3^b^^,^ c	25 ± 2^a^	1353 ± 74^a^	39 ± 2^a^	103 ± 2^a^	38 ± 2^a^
40	165 ± 10^a^^,^ b ^,^ c	73 ± 3^b^^,^ c	29 ± 2^a^	1990 ± 81^a^	55 ± 2^a^	102 ± 1^a^	40 ± 1^a^
60	179 ± 12^a^	74 ± 3^b^	32 ± 2^a^	2779 ± 72^a^^,^ c	77 ± 3^a^^,^ c	106 ± 1	40 ± 1^a^
80	192 ± 11^a^^,^ b ^,^ c	79 ± 3^b^^,^ c	41 ± 2^a^	3684 ± 110^a^^,^ b ^,^ c	110 ± 5^a^^,^ b ^,^ c	111 ± 2	38 ± 1^a^
100	211 ± 9^a^^,^ b ^,^ c	84 ± 3^b^^,^ c	51 ± 3^a^	4422 ± 83^a^^,^ b ^,^ c	148 ± 7^a^^,^ b ^,^ c	115 ± 1^a^	34 ± 1
HYP_mild_							
Rest	135 ± 5^a^^,^ b	72 ± 4^b^	16 ± 2	392 ± 19	14 ± 1	108 ± 2	34 ± 1
20	162 ± 6^a^^,^ b	76 ± 4^a^^,^ b	25 ± 2^a^	1138 ± 744^a^	39 ± 2^a^	100 ± 2^a^	39 ± 1^a^
40	182 ± 8^a^	80 ± 3^a^	29 ± 2^a^	2124 ± 73^a^	58 ± 2^a^	102 ± 1^a^	40 ± 1^a^
60	196 ± 12^a^	81 ± 5^a^^,^ b	34 ± 2^a^	3052 ± 92^a^^,^ b	86 ± 3^a^^,^ b	106 ± 1	40 ± 1^a^
80	211 ± 11^a^	86 ± 5^a^	41 ± 2^a^^,^ b	4102 ± 103^a^^,^ b	126 ± 4^a^^,^ b	113 ± 1	36 ± 1^a^
100	229 ± 11^a^	96 ± 6^a^	52 ± 3^a^	4733 ± 158^a^	161 ± 7^a^	116 ± 1^a^	34 ± 1
Control							
Rest	155 ± 6^c^	83 ± 4	17 ± 2	404 ± 27	14 ± 1	108 ± 3	33 ± 1
20	180 ± 4^a^^,^ c	85 ± 3	26 ± 2^a^	1332 ± 75^a^	39 ± 2^a^	99 ± 1^a^	38 ± 1^a^
40	200 ± 7^a^	87 ± 3	29 ± 2^a^	2058 ± 80^a^	57 ± 2^a^	102 ± 2^a^	40 ± 1^a^
60	217 ± 7^a^	91 ± 3^a^	32 ± 2^a^	2878 ± 84^a^	79 ± 3^a^	104 ± 2	40 ± 1^a^
80	227 ± 7^a^	92 ± 4^a^	39 ± 2^a^	3882 ± 114^a^	116 ± 6^a^	110 ± 2	38 ± 1^a^
100	245 ± 8^a^	100 ± 5^a^	52 ± 3^a^	4729 ± 124^a^	165 ± 7^a^	117 ± 1^a^	33 ± 1

Values are mean ± SEM for nine participants. Presented symbols denote differences between conditions, when compared at a relative % of maximal work rate in hyperthermia or normothermia (100% = 321 W in HYP_mod_ vs. 371 W in HYP_mild_ and control, respectively).

HR, Heart rates; SBP, systolic blood pressure; DBP, diastolic blood pressure; r*f,* respiratory frequency; *V̇*CO_2,_carbon dioxide production; *V̇*_E,_ minute ventilation; PetO_2,_ end‐tidal oxygen; PetCO_2_, carbon dioxide tension.

a Different versus rest *P *<* *0.05.

b Different versus control.

c Different versus mild hyperthermia.

### Brain, active limb, and systemic hemodynamics

**Figure 4:**
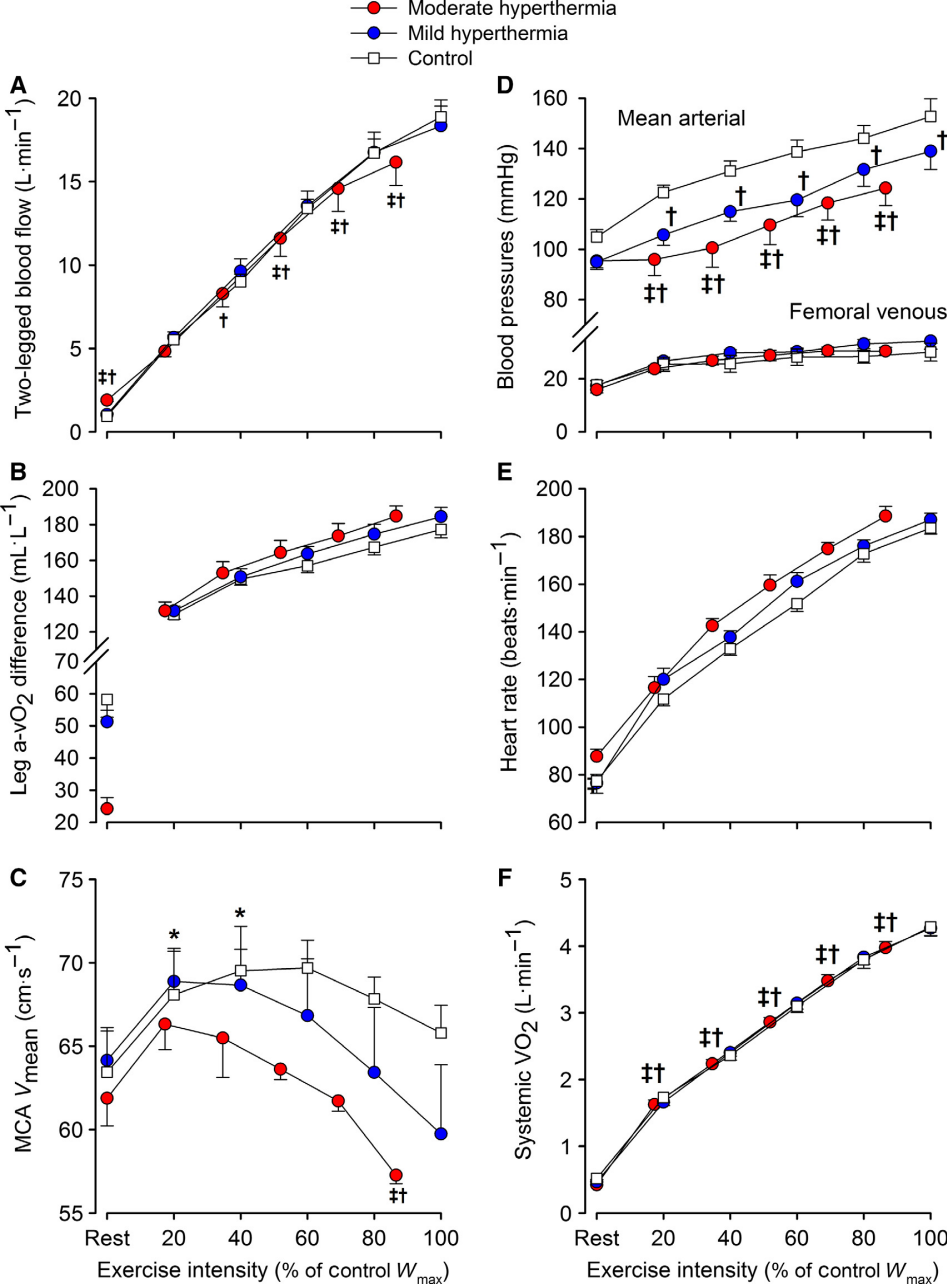
Two‐legged and brain hemodynamics, blood pressures and systemic oxygen uptake in response to exercise with different grades of hyperthermia. Values are means ± SEM for nine participants. Variables in all figures (except Fig. 4C) increased with exercise intensity. Limb blood flow (Fig. 4A) increased with exercise intensity to ~80% *W*_max_ (*P *<* *0.05), but plateaued prior to exhaustion. ^‡^different versus mild hyperthermia, ^†^different versus control. Presented symbols denote differences between conditions, when compared at a relative % of maximal work rate in hyperthermia or normothermia (100% = 321 W in HYP_mod_ vs. 371 W in HYP_mild_ and control, respectively).

At baseline, HR was 57 ± 3 beats·min^−1^, two‐legged blood flow 0.8 ± 0.1 L·min^−1^, *Q̇* 5.5 ± 0.4 L·min^−1^, and MCA *V*_mean_ 64 ± 1 cm·s^−1^ (Fig. 4). At rest following passive heat stress or control, HR (88 ± 3 vs. ~76 ± 5 bpm), two‐legged blood flow (1.9 ± 0.1 vs. ~1.0 ± 0.1 L·min^−1^), and *Q̇* (8.9 ± 0.7 vs. ~6.9 ±0.8 L·min^−1^) were elevated in HYP_mod_ compared to HYP_mild_ and control (all *P *<* *0.05), whereas MCA *V*_mean_ was not different (~63 ± 2 cm·s^−1^). From rest to submaximal exercise, HR and two‐legged blood flow increased with exercise intensity in all conditions (*P *<* *0.05 vs. rest) and MCA *V*_mean_ was elevated (Fig. 4C; *P *<* *0.05). However, overall, two‐legged blood flow was lower (Fig. 4A; *P *<* *0.05) and HR higher, in HYP_mod_ exercise compared to control exercise. At exhaustion, HR increased to similar peak values in HYP_mod,_ HYP_mild_ and control, respectively (189 ± 4, 187 ± 3, and 184 ± 3 beats·min^−1^). In all conditions, the rate of rise in two‐legged blood flow was attenuated, and MCA *V*_mean_ was reduced in all exercise conditions. Final two‐legged blood flow (16.2 ± 1.3, 18.4 ± 1.1, and 18.9 ± 1.1 L·min^−1^) and MCA *V*_mean_ (57 ± 1 vs. 66 ± 3 cm·s^−1^) were lower in lower in HYP_mod_ than in HYP_mild_ and control conditions.

On the transition from rest to submaximal exercise, estimated *Q̇* increased at a similar rate among conditions (~0.04 L·min^−1^·W^−1^). Prior to exhaustion, *Q̇* paralleled the attenuation in two‐legged blood flow, to a greater extent in HYP_mod_ versus HYP_mild_ and control conditions (Gradient = 0.007 vs. 0.017 L·min^−1^·W^−1^), at a lower absolute work rate, and was similar at end‐exercise (26.6 ± 2 L·min^−1^).

### Blood pressure, oxygen uptake, and brain oxygenation

At rest, MAP and FVP were not different among conditions (Fig. 4D). From rest to maximal exercise, MAP increased in all conditions, but was reduced in HYP_mod_ compared to HYP_mild_ and control, respectively (124 ± 7, 139 ± 7, and 153 ± 7 mmHg; *P *<* *0.05). Femoral venous pressure increased with exercise intensity but was not different among exercise conditions.

At rest, leg a‐v O_2diff_ was lower in HYP_mod_ compared to HYP_mild_ (24 ± 3 vs. ~56 ± 7 mL·L^−1^; *P *<* *0.05: Fig. 4B), whereas resting systemic *V̇*O_2_ was not different among conditions (0.46 ± 0.03 L·min^−1^; *P* = 0.47–0.84). During incremental exercise, leg a‐vO_2diff_ and systemic *V̇*O_2_ increased with intensity in all conditions (*P *<* *0.05). At exhaustion, leg a‐v O_2diff_ was not different among conditions; however, systemic *V̇*O_2max_ was reduced in HYP_mod_ compared to HYP_mild_ and control exercise conditions (3.94 ± 0.11 vs. 4.23 ± 0.13 and 4.23 ± 0.14 L·min^−1^, respectively; *P *<* *0.05). Compared to the three maximal incremental tests in the control trial, the rise in systemic *V̇*O_2_ per unit of power was identical from 20 to 70–80%*W*_max_ (9.6 ± 0.3 mL·min^−1^·W^−1^), but became attenuated thereafter (8.2 ± 0.6 mL·min^−1^·W^−1^). At rest, NIRS‐derived rSO_2_% was elevated in HYP_mod_ and HYP_mild_ versus control conditions (77 ± 2 & 75 ± 3 vs. 67 ± 3%; *P *<* *0.05) and remained unchanged across all conditions during incremental exercise, but declined before exhaustion (rSO_2_ ~64%; *P *<* *0.05).

### Brain and active limb conductance, blood gasses, plasma catecholamines and ATP

Arterial and venous hemoglobin [Hb] and arterial oxygen content increased with incremental exercise in all conditions, despite a reduction in arterial oxygen saturation (all *P *<* *0.05: Table 2 and 3). Arterial oxygen content was elevated in both heat stress conditions and was higher in HYP_mod_ compared to HYP_mild_ and control up to 60% W_max_ (*P *<* *0.05). Blood lactate increased exponentially and reached similar values at exhaustion in all experimental conditions (Table 3). However, arterial and venous glucose concentrations were elevated at exercise intensities ≥ 60% W_max_ in the HYP_mod_ compared to control and HYP_mild_.

**Table 2:** Blood gasses and metabolite responses to incremental exercise with different grades of hyperthermia

% of W_max_	pH		Hb (g·L^−1^)		*S*O_2_ (%)		*P*O_2_ (mmHg)		*P*CO_2_ (mmHg)	
	Arterial	Venous	Arterial	Venous	Arterial	Venous	Arterial	Venous	Arterial	Venous
HYP_mod_										
Rest	7.46 ± 0.01^b^^,^ b	7.44 ± 0.01^b^^,^ b	148 ± 3^b^^,^ b	151 ± 3^b^^,^ b	97.5 ± 0.3	85.1 ± 1.3^b^^,^ b	94.5 ± 2.9	51.8 ± 1.3^b^^,^ b	38.2 ± 1.3	42.1 ± 1.8
20	7.47 ± 0.01^b^^,^ [Link]	7.39 ± 0.01^a^^,^ b ^,^ [Link]	154 ± 3^a^^,^ b ^,^ [Link]	157 ± 3^a^^,^ b ^,^ [Link]	98.0 ± 0.2	36.3 ± 1.2^a^	100.3 ± 2.8	24.1 ± 0.6^a^^,^ b ^,^ [Link]	36.2 ± 1.8^a^	53.7 ± 2.8^a^
40	7.45 ± 0.01^a^^,^ b ^,^ [Link]	7.35 ± 0.01^a^	154 ± 3^a^^,^ b ^,^ [Link]	157 ± 3^a^^,^ b ^,^ [Link]	97.8 ± 0.2	25.9 ± 2.0^a^	99.4 ± 3.1	21.1 ± 0.8^a^	37.0 ± 1.5[Link]	60.3 ± 2.7^a^
60	7.42 ± 0.01^a^^,^ b	7.31 ± 0.01^a^	155 ± 3^a^^,^ b ^,^ [Link]	158 ± 3^a^^,^ b ^,^ [Link]	97.5 ± 0.2^a^	21.0 ± 2.3^a^	99.3 ± 2.2	20.2 ± 1.1^a^	38.1 ± 1.2	65.9 ± 2.1^a^
80	7.40 ± 0.01^a^^,^ b ^,^ [Link]	7.26 ± 0.01^a^^,^ [Link]	156 ± 3^a^	159 ± 4^a^^,^ b ^,^ [Link]	97.2 ± 0.2^a^	16.8 ± 1.9^a^^,^ [Link]	98.2 ± 2.9	18.9 ± 1.1^a^	36.2 ± 0.9	72.1 ± 1.9^a^
100	7.36 ± 0.01^a^^,^ b ^,^ [Link]	7.19 ± 0.01^a^^,^ [Link]	157 ± 3^a^	161 ± 3^a^^,^ b ^,^ [Link]	96.7 ± 0.2^a^	11.6 ± 1.3^a^	100.3 ± 2.4	17.8 ± 1.1^a^	33.7 ± 1.0^a^	78.1 ± 2.1^a^
HYP_mild_										
Rest	7.44 ± 0.01	7.41 ± 0.01	141 ± 2^b^	143 ± 3	97.9 ± 0.1	71.5 ± 2.1	95.7 ± 2.2	38.2 ± 1.2	38.2 ± 1.0	44.3 ± 1.2
20	7.44 ± 0.01	7.38 ± 0.01^a^	147 ± 3^a^^,^ b	149 ± 3^a^^,^ b	97.8 ± 0.1	32.4 ± 1.6^a^	95.1 ± 2.0	21.8 ± 0.5^a^	37.7 ± 1.1	52.5 ± 1.6^a^
40	7.42 ± 0.01^a^	7.33 ± 0.01^a^	148 ± 3^a^^,^ b	150 ± 3^a^^,^ b	97.6 ± 0.2	23.4 ± 1.2^a^	95.9 ± 1.8	20.0 ± 0.5^a^	39.3 ± 0.9	61.4 ± 1.5^a^
60	7.41 ± 0.00^a^	7.29 ± 0.00^a^	150 ± 3^a^^,^ b	153 ± 3^a^^,^ b	97.3 ± 0.1^a^	18.0 ± 1.2^a^	96.0 ± 1.7	18.6 ± 0.6^a^	38.6 ± 1.0	67.6 ± 1.2^a^
80	7.38 ± 0.01^a^	7.23 ± 0.01^a^^,^ b	153 ± 3^a^	155 ± 3^a^	97.1 ± 0.2^a^	13.9 ± 1.4^a^^,^ b	97.3 ± 2.0	17.8 ± 1.0^a^	36.6 ± 1.2^b^	74.0 ± 1.5^a^
100	7.32 ± 0.01^a^^,^ b	7.15 ± 0.01^a^^,^ b	156 ± 3^a^^,^ b	152 ± 3^a^^,^	96.2 ± 0.3^a^	11.1 ± 1.2^a^	99.2 ± 2.5	17.9 ± 1.2^a^	33.3 ± 1.1^a^	79.0 ± 2.7^a^
Control										
Rest	7.44 ± 0.01	7.41 ± 0.01	138 ± 3	140 ± 3	97.9 ± 0.1	66.6 ± 3.3	95.8 ± 1.6	36.4 ± 1.9	37.4 ± 1.0	43.9 ± 1.6
20	7.44 ± 0.01	7.39 ± 0.01^a^	145 ± 3^a^	146 ± 3^a^	97.7 ± 0.2	32.3 ± 1.2^a^	93.5 ± 2.1	21.7 ± 0.3^a^	37.2 ± 1.0	50.2 ± 1.5^a^
40	7.42 ± 0.00^a^	7.34 ± 0.01^a^	146 ± 3^a^	147 ± 3^a^	97.7 ± 0.2	23.0 ± 1.6^a^	97.5 ± 2.4	19.6 ± 0.6^a^	38.7 ± 1.0	59.5 ± 1.5^a^
60	7.40 ± 0.00^a^	7.29 ± 0.00^a^	148 ± 3^a^	151 ± 3^a^	97.2 ± 0.2^a^	19.7 ± 1.6^a^	95.2 ± 1.8	19.2 ± 0.8^a^	39.4 ± 1.0^a^	66.0 ± 1.4^a^
80	7.38 ± 0.01^a^	7.24 ± 0.01^a^	150 ± 3^a^	151 ± 3^a^	96.8 ± 0.2^a^	15.5 ± 1.5^a^	95.6 ± 2.5	18.6 ± 1.1^a^	38.3 ± 1.4	71.4 ± 1.6^a^
100	7.33 ± 0.01^a^	7.17 ± 0.01^a^	153 ± 3^a^	153 ± 3^a^	96.3 ± 0.3^a^	12.0 ± 1.2^a^	98.8 ± 2.6	17.9 ± 1.1^a^	34.3 ± 1.3^a^	76.7 ± 2.2^a^

Values are mean ± SEM for 9 participants. pH, Hemoglobin (Hb), oxygen saturation (*S*O_2_%), partial pressures of oxygen (*P*O_2_) and carbon dioxide (*P*CO_2_) for arterial and femoral venous blood.

Presented symbols denote differences between conditions, when compared at a relative % of maximal work rate in hyperthermia or normothermia (100% = 321 W in HYP_mod_ vs. 371 W in HYP_mild_ and control, respectively).

a Different versus rest.

b Different versus control (all *P *<* *0.05).

Different versus mild hyperthermia.

**Table 3:** Blood gasses and metabolite responses to incremental exercise with different grades of hyperthermia

% of W_max_	*Ct*O_2_ (mL·L^−1^)		[Lac] (mmol·L^−1^)		[Glu] (mmol·L^−1^)		[HCO_3_^−^] (mmHg)		ABE (mmol·L^−1^)	
	Arterial	Venous	Arterial	Venous	Arterial	Venous	Arterial	Venous	Arterial	Venous
HYP_mod_										
Rest	199 ± 4^b^^,^ c	176 ± 3^b^^,^ c	1.0 ± 0.1	1.1 ± 0.1	5.8 ± 0.1	5.8 ± 0.2	27.3 ± 0.3^b^^,^ c	27.7 ± 0.3^b^^,^ c	3.0 ± 0.4^b^^,^ c	4.1 ± 0.5^b^
20	209 ± 4^a^^,^ b ^,^ c	79 ± 2^a^^,^ b ^,^ c	1.7 ± 0.2^a^	2.1 ± 0.3^a^	5.9 ± 0.2	5.8 ± 0.3	26.6 ± 0.3^a^^,^ b	27.5 ± 0.4^b^	2.0 ± 0.5^a^^,^ b	6.5 ± 0.5^a^^,^ b
40	208 ± 4^a^^,^ b ^,^ c	56 ± 4^a^	2.2 ± 0.3^a^	2.4 ± 0.4^a^	6.0 ± 0.2	6.0 ± 0.3	26.1 ± 0.5^a^^,^ b	27.0 ± 0.5^a^	1.5 ± 0.6^a^	6.8 ± 0.6^a^^,^ b
60	209 ± 4^a^^,^ b ^,^ c	46 ± 5^a^	3.0 ± 0.4^a^	3.3 ± 0.5^a^	6.1 ± 0.2^b^^,^ c	6.0 ± 0.3^b^^,^ c	25.2 ± 0.5^a^^,^ b	26.0 ± 0.6^a^^,^ b	0.5 ± 0.7^a^	6.1 ± 0.8^a^^,^ b
80	210 ± 4^a^	37 ± 4^a^	4.8 ± 0.5^a^	5.4 ± 0.5^a^	6.1 ± 0.3^b^^,^ c	6.0 ± 0.3^b^^,^ c	23.3 ± 0.5^a^^,^ b ^,^ c	23.8 ± 0.6^a^^,^ b ^,^ c	−1.9 ± 0.7^a^^,^ c	4.2 ± 0.8^b^^,^ c
100	210 ± 4^a^	26 ± 3^a^	8.6 ± 0.6^a^	9.7 ± 0.5^a^	6.3 ± 0.3^a^^,^ b ^,^ c	6.3 ± 0.3^a^^,^ b ^,^ c	20.1 ± 0.6^a^^,^ b ^,^ c	20.3 ± 0.5^a^^,^ b ^,^ c	−6.1 ± 0.8^a^^,^ b ^,^ c	0.4 ± 0.7^a^
HYP_mild_										
Rest	191 ± 3^b^	140 ± 6	1.3 ± 0.2	1.5 ± 0.1	5.9 ± 0.2	5.7 ± 0.3	26.0 ± 0.3^b^	26.4 ± 0.3	1.7 ± 0.4^b^	3.4 ± 0.4
20	198 ± 4^a^^,^ b	67 ± 4^a^	1.5 ± 0.2^a^	1.6 ± 0.2	5.9 ± 0.3	5.8 ± 0.3	26.0 ± 0.3	26.7 ± 0.3	1.6 ± 0.4	5.6 ± 0.4^a^
40	199 ± 4^a^^,^ b	49 ± 2^a^	1.7 ± 0.2^a^	2.0 ± 0.3^a^	5.7 ± 0.3^a^	5.6 ± 0.3	25.7 ± 0.3^a^	26.3 ± 0.3	1.3 ± 0.4^a^	6.0 ± 0.5^a^
60	202 ± 4^a^^,^ b	38 ± 3^a^	2.8 ± 0.3^a^	3.3 ± 0.4^a^	5.4 ± 0.2	5.3 ± 0.2	24.7 ± 0.3^a^	25.2 ± 0.4^a^	−0.1 ± 0.5^a^	5.3 ± 0.5^a^
80	205 ± 4^a^	30 ± 3^a^	5.7 ± 0.6^a^	6.3 ± 0.7^a^	5.3 ± 0.2^a^	5.2 ± 0.3	22.2 ± 0.5[Link] ^,^ b	22.5 ± 0.5^a^	−3.1 ± 0.7^a^^,^ b	2.7 ± 0.7
100	208 ± 4^a^	23 ± 2^a^	10.5 ± 0.8^a^	11.0 ± 0.8^a^	5.3 ± 0.3	5.2 ± 0.3	18.3 ± 0.5^a^^,^ b	18.8 ± 0.5^a^	−8.3 ± 0.7^a^^,^ b	−1.8 ± 0.8^a^
Control										
Rest	187 ± 4	129 ± 9	1.4 ± 0.2	1.7 ± 0.2	6.1 ± 0.2	5.9 ± 0.1	25.5 ± 0.2	26.0 ± 0.3	1.0 ± 0.3	3.0 ± 0.5
20	195 ± 4^a^	65 ± 3^a^	1.6 ± 0.2	1.7 ± 0.2	6.0 ± 0.1^a^	6.1 ± 0.1	25.5 ± 0.3	26.4 ± 0.3	1.0 ± 0.4	4.9 ± 0.5^a^
40	197 ± 4^a^	47 ± 4^a^	1.7 ± 0.3	2.0 ± 0.3	5.9 ± 0.1	5.9 ± 0.2	25.4 ± 0.3	26.0 ± 0.4	0.9 ± 0.5	5.4 ± 0.6^a^
60	198 ± 4^a^	41 ± 4^a^	2.6 ± 0.3^a^	3.2 ± 0.4^a^	5.6 ± 0.2^a^	5.5 ± 0.2^a^	24.5 ± 0.4^a^	24.9 ± 0.5^a^	−0.1 ± 0.5^a^	4.8 ± 0.6^a^
80	201 ± 4^a^	33 ± 3^a^	4.8 ± 0.5^a^	5.5 ± 0.6^a^	5.3 ± 0.2^a^	5.2 ± 0.3^a^	22.7 ± 0.5^a^	22.8 ± 0.6^a^	−2.4 ± 0.8^a^	2.7 ± 0.8
100	203 ± 4^a^	26 ± 3^a^	9.3 ± 0.6^a^	10.3 ± 0.9^a^	5.2 ± 0.3^a^	5.1 ± 0.3^a^	19.1 ± 0.5^a^	19.4 ± 0.6^a^	−7.2 ± 0.8^a^	−0.8 ± 0.8^a^

Values are mean±SEM for nine participants. Oxygen content (*Ct*O_2_), lactate concentration ([Lac]), glucose concentration ([Glu]), sodium bicarbonate concentration ([HCO3^−^])), and acid‐base excess (ABE) for arterial and femoral venous blood.

Presented symbols denote differences between conditions, when compared at a relative % of maximal work rate in hyperthermia or normothermia (100% = 321 W in HYP_mod_ vs. 371 W in HYP_mild_ and control, respectively).

a Different versus rest *P *<* *0.05.

b Different versus control.

c Different versus mild hyperthermia.

**Figure 5:**
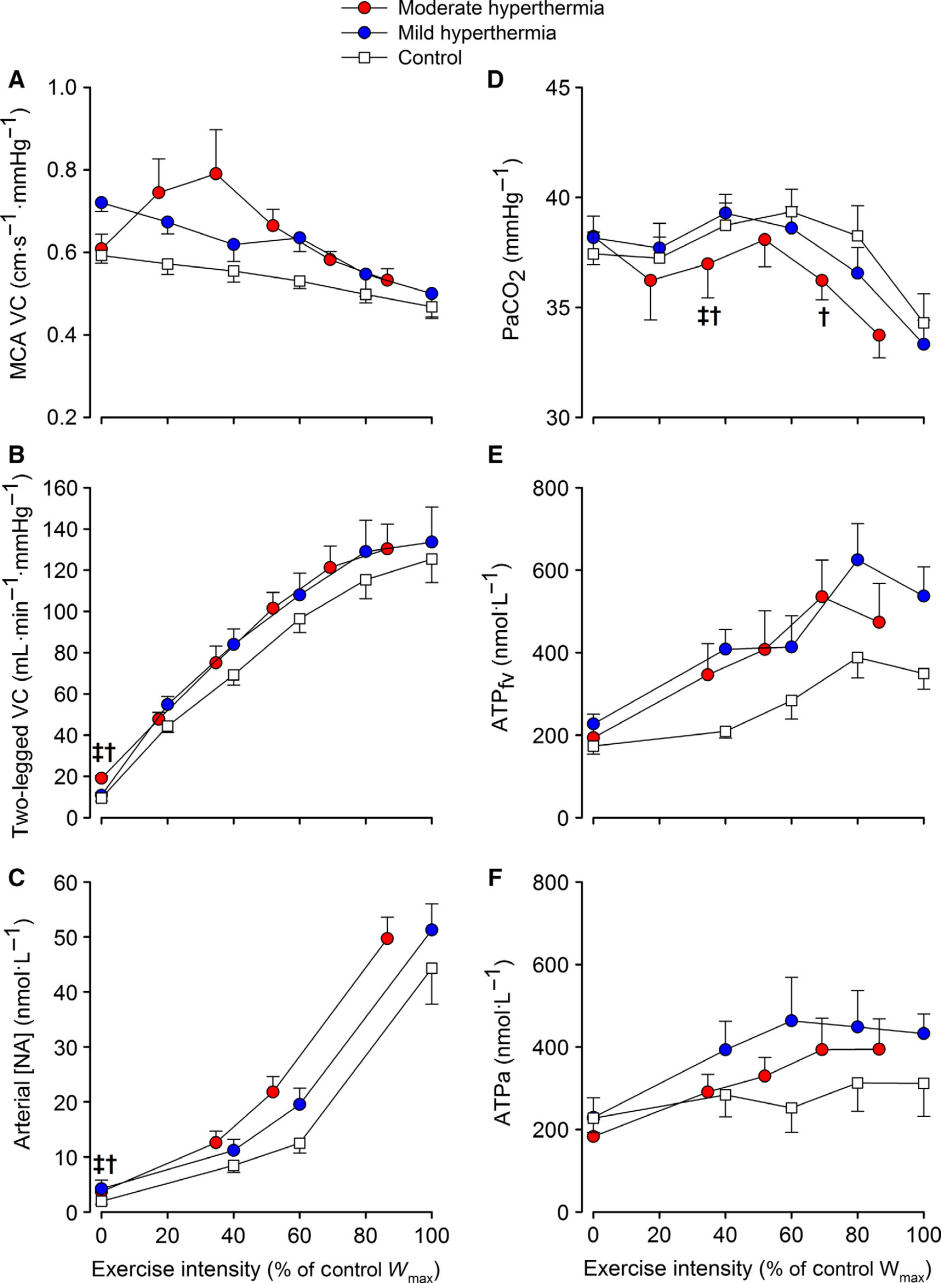
Brain and two‐legged vascular conductances, arterial [NA], *P*_a_ CO_2_ and femoral venous and arterial plasma ATP in response to incremental exercise with different grades of hyperthermia. Values are means±SEM for seven participants. ^‡^different versus mild hyperthermia, ^†^different versus control. Presented symbols denote differences between conditions, when compared at a relative % of maximal work rate in hyperthermia or normothermia (100% = 321 W in HYP_mod_ vs. 371 W in HYP_mild_ and control, respectively).

At rest, MCA vascular conductance was not different among conditions (Fig. 5A). The elevations in limb and systemic perfusion in HYP_mod_ were coupled to an enhanced limb vascular conductance (Fig. 5B; *P *<* *0.05). MCA vascular conductance declined with exercise intensity. Contrastingly, limb vascular conductance increased with exercise intensity (*P *<* *0.05), but was not different among conditions.

At rest, arterial [NA] (Fig. 5C) was augmented in HYP_mod_ versus HYP_mild_ and control conditions (3.7 ± 0.8 vs. ~1.9 ± 0.5 nmol·L^−1^; *P *<* *0.05), whereas venous [NA] was not different (data not shown). Thereafter, both arterial and venous [NA] increased with exercise intensity to a similar peak value (~48 ± 5 nmol·L^−1^). The rise in arterial [NA] was coupled to a blunted two‐legged vascular conductance at maximal exercise intensities (*R* ^*2*^ = 0.64; *P *<* *0.01). At rest and during submaximal exercise, *P*_a_CO_2_ was maintained stable (Fig. 5D. However, beyond submaximal intensities, and in association with a marked increase in *V̇*_E_ (Table 1), *P*_a_CO_2_ declined to a similar end‐exercise value across conditions (*P *<* *0.05). The decline in *P*_a_CO_2_ was moderately related to the fall in MCA vascular conductance (*R* ^*2*^ = 0.29; *P *<* *0.01). Lastly, femoral venous (ATP_fv_) and arterial (ATP_a_) ATP concentrations were not different at rest, but increased up to submaximal exercise intensities in all conditions (Fig. 5E and F). Beyond 80% *W*_max_, both ATP_fv_, in association with an attenuation in two‐legged vascular conductance (*R* ^*2*^ = 0.46; *P *<* *0.01), and ATP_a_ plateaued.

## Discussion

To the best of our knowledge, this is the first study to separate the effects of skin hyperthermia from the combined effects of skin and internal hyperthermia on the brain and locomotor limb perfusion, aerobic metabolism and exercise capacity. The major novel finding was that marked skin hyperthermia was insufficient to compromise *V̇*O_2max_ and incremental exercise capacity. On the other hand, superimposed internal and skin hyperthermia reduced maximal exercise capacity and brain and locomotor limb perfusion, which were mechanistically coupled to a plateau or decline in regional vascular conductance and a reduced arterial pressure. Finally, the attenuation in the brain and exercising limb blood flow appears to be an important mechanism by which combined skin and internal hyperthermia reduces aerobic metabolism and exercise capacity. Together, these findings demonstrate that the combination of skin and internal body hyperthermia is a critical factor in whether, or not, brain and active muscle perfusion and aerobic metabolism is compromised during incremental exercise to volitional exhaustion in hot environments.

### Skin hyperthermia does not independently compromise cardiovascular capacity or aerobic exercise performance

In this study, we clamped skin temperature at a high level (i.e., ~37°C vs. ~32°C), without increasing internal temperature, prior to and during incremental exercise (HYP_mild_). To achieve this, the participants were first exposed to passive whole body heat stress for ~13 min and then combined whole body heat stress and exercise for an additional ~12.5 min. An important finding under these conditions was that *V̇*O_2max_ and exercise capacity was the same compared to control exercise. Narrow core‐to‐skin temperature gradients, as seen in both HYP conditions in this study (range: 0–2.6°C), are purported to place a significant burden on cardiovascular capacity owing to the increased demand for skin blood flow (Rowell 1986; Sawka et al. 2012). This theory has been taken to mean that high skin temperatures play a dominant role in reduced exercise capacity in the heat, by promoting the displacement of blood volume and flow to the skin thereby compromising active muscle perfusion (Tatterson et al. 2000; Ely et al. 2009; Kenefick et al. 2010; Lorenzo et al. 2010; Cheuvront et al. 2010; Sawka et al. 2012). However, we show that brain, active limb, and systemic blood flow during HYP_mild_ is not reduced compared to that observed during control conditions. Moreover, the results on the experimental trial are supported by those on the control trial (Fig. 2) where despite some differences in exercise internal temperature (~0.5°C at exhaustion), exercise capacity was not different during repeated incremental exercise with normal skin temperature (~32°C). Our findings collectively suggest that skin hyperthermia or small elevations in internal temperature alone do not compromise aerobic power or exercise capacity in trained individuals.

In contrast, when combined internal and skin hyperthermia was present (i.e., achieved by extending the exposure to passive whole body heat stress to ~52 min, while the exercise duration was not different), *V̇*O_2max_ was reduced by ~8%; a decline similar to that previously reported (Klausen et al. 1967; Pirnay et al. 1970; Sawka et al. 1985; Nybo et al. 2001; Arngrímsson et al. 2004). The reduced aerobic power and work capacity were associated with a diminished arterial pressure, an attenuation in active limb (and systemic perfusion), a reduced brain blood flow, and high internal and skin temperatures (39.3 and 37°C, respectively) (Fig. 4). Restricted LBF, via a plateau in local vascular conductance, precedes fatigue during incremental (Mortensen et al. 2008) and constant load maximal exercise (González‐Alonso and Calbet 2003); whole body hyperthermia advances this cardiovascular instability and may explain the reduced maximal aerobic power (González‐Alonso and Calbet 2003). Our data demonstrate that the duration of heat exposure is critical to whether or not cardiovascular function is impaired during strenuous exercise in the heat‐stressed human.

### Impact of hyperthermia on blood flow and pressure at rest and during incremental exercise

To understand the responses to regional hyperthermia during exercise and the potential underlying mechanisms, we need to first scrutinize the resting responses. At rest, combined internal and skin hyperthermia led to elevations in LBF and *Q̇*, accompanying a fall in limb a‐vO_2diff_ and a lower MAP, in close agreement with the responses to passive heat stress (Barcroft et al. 1947; Rowell et al. 1969; Rowell 1974; Minson et al. 1998; Crandall et al. 2008; Stöhr et al. 2011a; Pearson et al. 2011; Heinonen et al. 2011; Chiesa et al. 2016). Interestingly, brief heat exposure, sufficient to raise *T*_sk_ to that experienced during combined internal and skin hyperthermia (HYP_mild_), but without elevations in *T*_c_, led to a small increase in systemic (+1.3 L·min^−1^) and limb blood flow (+0.25 L·min^−1^) compared to baseline values. During passive whole body heat stress, interspersed by single leg exercise, elevations in whole‐body perfusion (e.g., *Q̇*; 1.1–1.8 ± 0.3 L·min^−1^, LBF; 0.5 ± 0.1 L·min^−1^) and small but significant reductions in MAP have been observed with skin hyperthermia at rest without increases in *T*_c_ (Pearson et al. 2011; Stöhr et al. 2011a). In a recent study from this laboratory, mild heat stress was also shown to induce small but significant increases in systemic and leg perfusion and HR (e.g., *Q̇*; 0.9 L·min^−1^, LBF; 0.2 L·min^−1^; 12 beats·min^−1^), although these alterations occurred concomitant to small increases in *T*_c_ (~0.4°C) (Chiesa et al. 2016). It is therefore likely that any increased demand for skin and deep limb tissue blood flow, during passive mild hyperthermia, is met by blood flow redistribution from splanchnic vascular beds and a small increase in *Q̇* and small reduction in MAP (Rowell et al. 1968; Crandall et al. 2008).

A key question is whether passive hyperthermia‐induced hyperperfusion and hypotension alters cardiovascular dynamics during incremental exercise. There were no clear differences in hemodynamic responses, among conditions, during submaximal exercise; in agreement with previous blood flow observations in trained individuals (Savard et al. 1988; Nielsen et al. 1993, 1997). However, at the maximal attainable aerobic work rate in HYP_mod_, blood pressure, brain perfusion, and two‐legged blood flow were reduced compared to control exercise (Fig. 4), despite an estimated ~1.8 L·min^−1^ elevation in *Q̇* and a similar HR_max_ and limb O_2_ extraction. The reduced regional blood flow was coupled to an attenuation of vascular conductance, and enhanced vasoconstrictor activity (Fig. 5). Attenuation in blood flow and vascular conductance at maximal exercise may involve the interaction of various reflex, chemical, and thermal mechanisms, in different tissues of the body, responsible for regulating local vascular tone (Rowell 1974; González‐Alonso et al. 2004; González‐Alonso 2012; Mortensen et al. 2008; Mortensen and Saltin 2014). To provide mechanistic insight into these circulatory alterations during incremental exercise, with differing combinations of body temperatures, we assessed several vasoactive substances implicated in the regulation of the brain and muscle blood flow. Irrespective of the temperature manipulation, the suppressed (brain) or nonlinear rise in (two‐legged) blood flow prior to volitional exhaustion was coupled to a similar fall or plateau in regional vascular conductance (Fig. 5), indicative of vasoconstriction in the active brain and muscle vascular beds. Mechanistically, the brain blood flow velocity decline toward baseline values was associated with a hyperventilation‐induced fall in *P*_a_CO_2_ (*r* = 0.54; *P *<* *0.05); a potent vasoactive substance affecting cerebrovascular tone (Willie et al. 2012). These dynamics during graded exercise are supported by the literature (Hellstrom et al. 1996; Sato et al. 2011; Trangmar et al. 2014). On the other hand, the restriction in two‐legged conductance, prior to exhaustion in all conditions, was related to a plateau in plasma ATP and an exponential rise in sympathetic vasoconstrictor activity even when leg vascular conductance and plasma ATP and [NA] were or tended to be higher in the hyperthermic trials (Fig. 5). It has previously been postulated that the influence of sympathetic vasoconstriction on vascular conductance can be “overridden” by metabolic vasodilation (Remensnyder et al. 1962; Rosenmeier et al. 2004). This theory can explain the regulation of muscle perfusion when exercising limb blood flow, and the intravascular vasodilator milieu including ATP, increase progressively during exercise against a background of relatively low sympathetic drive (González‐Alonso et al. 2002; Rosenmeier et al. 2004; Mortensen et al. 2011). However, the present findings together with those during maximal and supramaximal exercise (Mortensen et al. 2008), indicate that local vasoconstriction prevails during whole body, intense exercise in association with marked increases in sympathetic nerve activity (Saito et al. 1993; Ichinose et al. 2008) and a blunted rise in plasma ATP concentration. Thus, functional sympatholysis does not prevail at the maximal and supramaximal exercise domain with normal or elevated levels of local hyperthermia.

### Does cardiovascular strain contribute to hyperthermia‐induced fatigue?

An important question from this study is which cardiovascular process underpins the reduced exercise capacity under physiologically stressful environments. Prevailing theory suggests that reduced aerobic capacity during exercise in the heat is due to reductions in active muscle blood flow, secondary to a substantial increase in skin perfusion, and despite active redistribution of blood flow from nonactive tissues (Rowell 1974, 1986). This theory was based on observations that body hyperthermia suppressed *Q̇* during treadmill running, in untrained and unacclimatized individuals, compared to control conditions (Rowell et al. 1966); thus giving rise to the premise that the limited cardiovascular capacity is insufficient to meet the combined demands of heat dissipation (skin perfusion) and active muscle perfusion. Our findings demonstrate that the attenuated rise in systemic *V̇*O_2_ (from 9.6 to 8.2 mL·min^−1^·W^−1^) and reduced exercise capacity with combined internal and skin hyperthermia were coupled to an advanced fall in brain blood flow, and an early attenuation in LBF (that is, occurring at a lower absolute work rate); temporal responses that could feasibly result in a compromised local tissue aerobic metabolism when oxygen extraction reaches its upper limits (~ 90% in the three conditions of this study) (González‐Alonso and Calbet 2003; Mortensen et al. 2005, 2008; Calbet et al. 2007). In addition, our estimates of *Q̇* suggest that systemic blood flow is similar at exhaustion among temperature manipulations; a conclusion supported by findings in trained participants, during constant‐load cycling to volitional exhaustion, with combined internal and skin hyperthermia (González‐Alonso and Calbet 2003). It is therefore unlikely that the absolute values of *Q̇* and high skin blood flow explain early fatigue during incremental exercise.

Reductions in cerebral O_2_ delivery (and oxygenation) might contribute to fatigue processes when hyperthermic (Nielsen and Nybo 2003; Nybo and Secher 2004; Todd et al. 2005; Rasmussen et al. 2010; Ross et al. 2012). However, it is unlikely that the moderate reductions in cerebral perfusion, seen here, and in previous studies (González‐Alonso et al. 2004; Trangmar et al. 2014, 2015), can compromise cerebral metabolism to the extent that can explain the reduced aerobic power with a moderate hyperthermia. Rather, the advanced fall in cerebral perfusion, at lower absolute exercise intensities, is likely a consequence of the overall cardiovascular strain induced by strenuous exercise in the heat and the concomitant respiratory alkalosis. This is supported by similar findings in hypoxia where cerebral O_2_ delivery is markedly attenuated, despite elevated systemic blood flow and perfusion pressure (Subudhi et al. 2009; Vogiatzis et al. 2011). Restoring reductions in cerebral O_2_ delivery, during exercise in hypoxia and with body hyperthermia, does not improve maximal aerobic power (Subudhi et al. 2011; Keiser et al. 2015), indicating that processes other than a suppressed cerebral O_2_ metabolism explain the early fatigue under physiological stressful environments.

Our present findings highlight that combined skin and internal hyperthermia compromises regional and systemic perfusion at the maximal attainable aerobic work rate. Blunted skeletal muscle and systemic blood flow and O_2_ delivery, with and without body hyperthermia, appear to be an important factor limiting aerobic capacity (González‐Alonso and Calbet 2003; Mortensen et al. 2005, 2008). We recognize that many interrelating factors likely contribute to the development of fatigue during exercise. In this context, exhaustion in the present experimental conditions may have resulted from the interaction of multiple inhibitory and excitatory regulatory processes in response to reduced O_2_ delivery, modified locomotor muscle and brain metabolism, hyperthermia, altered central motor output, changed central nervous system neurotransmitter activity, and stimulation of muscle feedback mechanisms sensing local metabolic milieu (as widely reviewed; González‐Alonso et al. 2008; Amann and Calbet 2008; Meeusen and Roelands 2010; Amann et al. 2011; Noakes 2012; Sawka 2012; Nybo et al. 2014; Morales‐Alamo et al. 2015; Blain et al. 2016). Supporting the idea that the etiology of fatigue during exercise is multifactorial and typified by cardiovascular strain and disturbed physiological homeostasis, we found that the single stressor skin hyperthermia was apparently met by compensatory physiological adjustments such that muscle and whole body aerobic energy provision was not compromised compared to control. The combination of multiple stressors triggered by whole body hyperthermia, however, resulted in a compromised aerobic capacity, associated with a blunted active muscle and systemic perfusion.

### Methodological considerations

Resting blood flow measurements were made using Doppler ultrasonography, rather than thermodilution, as less blood flow variability is seen with ultrasonography in resting conditions. We were unable to obtain direct measures of *Q̇* during exercise; on this basis, our conclusions based on estimated *Q̇* are purposefully tempered. On the other hand, it is established that systemic O_2_ difference shares a strong linear relationship with leg O_2_ extraction during incremental exercise (Mortensen et al. 2008; Munch et al. 2014). Moreover, the adjustment to this relationship in HYP_mod_ is in accordance with previous literature demonstrating a reduced systemic O_2_ extraction, per unit of leg O_2_ extraction with body hyperthermia (González‐Alonso et al. 2004). Finally, our estimations on *Q̇* dynamics during exercise were supported by those obtained with the Modelflow method. Nevertheless, future studies measuring central hemodynamics with different manipulations of internal and skin temperature are required to confirm the present observations.

## Conclusion

The present findings show that skin hyperthermia, in the absence of high internal temperatures, does not compromise cardiovascular capacity, maximal oxygen uptake, or exercise performance during strenuous whole‐body dynamic exercise. The fall in maximal aerobic power with combined internal and skin hyperthermia was associated with compromised active muscle metabolism due to reduced oxygen delivery. Taken together, these observations explain why aerobic exercise performance in hot environments is not universally impaired across all exercise modalities, as the deleterious effects of environmental heat stress are directly dependent upon heat exposure inducing whole‐body hyperthermia and uncompensable physiological strain.

## Conflict of Interest

All authors ascertain no conflict of interests associated with this work.
